# Supplementary material for: Adherence to COVID-19 Nutrition Guidelines Is Associated with Better Nutritional Management Behaviors of Hospitalized COVID-19 Patients
Source: Nutrients. 2021 Jun 3;13(6):1918. doi: 10.3390/nu13061918 (PMC8226761; doi:10.3390/nu13061918)
Supplement: Supplementary file 1 [file nutrients-13-01918-s001.zip › nutrients-1192835-supplementary.pdf]

**Supplementary Table S1** Barriers to dietician adherence to clinical practice of nutrition guidelines for hospitalized COVID-19 patients

The questionnaire consisted of 4 domains with a total of 28 questions: knowledge (12 questions), attitudes (6 questions), environmental factors (7 questions), and behaviors (3 questions). Depending on the participant's answer, each question was awarded 1 or 0 points, with a maximum of 28 points in total. A higher score of knowledge, attitude, environment, and behavior indicates better adherence to nutritional practice guidelines for hospitalized COVID-19 patients. For example, 1 point was awarded to participants if they know "ESPEN guideline on clinical nutrition in the intensive care unit".

| Barrier | Score | |
|---|---|---|
| | Yes/Agree | No/Disagree |
| **Knowledge (Total score = 12)** | | |
| **Awareness of the guidelines (total score = 4)** | | |
| Do you know the following guidelines? | | |
| ESPEN guidelines on clinical nutrition in the intensive care unit. | 1 | 0 |
| ESPEN expert statements and practical guidance for nutritional management of individuals with SARS-CoV-2-infection (Europe). | 1 | 0 |
| Nutrition Therapy in the Patient with COVID-19 Disease Requiring ICU Care (reviewed and approved by the Society of Critical Care Medicine and the American Society for Parenteral and Enteral Nutrition). | 1 | 0 |
| Coronavirus Disease 2019 (COVID-19) Treatment Guidelines. National Institutes of Health. | 1 | 0 |
| **Familiarity with clinical practice (total score = 8)** | | |
| Nutritional assessment and early nutritional care management of COVID-19 patients must be integrated into the overall therapeutic strategy. | 1 | 0 |
| It is important to conduct nutritional screening and nutritional assessment of hospitalized COVID-19 patients. | 1 | 0 |
| It is important to monitor the body weight change of hospitalized COVID-19 patients. | 1 | 0 |
| It is important to monitor the dietary intake of hospitalized COVID-19 patients. | 1 | 0 |
| Nutrition therapy plays an important role in the outcomes of COVID-19 treatment. | 1 | 0 |
| r | 1 | 0 |
| Nutrition counseling is important for COVID-19 patients. | 1 | 0 |

| | | |
|---|---|---|
| Lack of clear guidelines. | 0 | 1 |
| **Attitudes (total score = 6)** | | |
| **Self-efficacy in performing nutrition care (Score = 3)** | | |
| I am knowledgeable about the role of nutrition therapy for COVID-19 patients. | 1 | 0 |
| Lack of self-efficacy or confidence in performing nutrition care for COVID-19 patients. | 0 | 1 |
| I have adequate knowledge to design meals for hospitalized COVID-19 patients with or without poly-comorbidities. | 1 | 0 |
| **Motivation in performing nutritional care (total score = 3)** | | |
| I regularly make decisions regarding nutrition therapy as part of the management of COVID-19 patients. | 1 | 0 |
| I have an obligation to improve the health of COVID-19 patients by discussing nutrition with them. | 1 | 0 |
| Feel stress when conducting nutrition therapy for COVID-19 patients (score = 1). | 0 | 1 |
| **Environmental factors (total score = 7)** | | |
| Lack of time. | 0 | 1 |
| Lack of resources. | 0 | 1 |
| Limited budget. | 0 | 1 |
| Limited food supplies. | 0 | 1 |
| Lack of access to meet COVID-19 patients in person. | 0 | 1 |
| Lack of access to medical records. | 0 | 1 |
| Inadequate authority to perform nutrition care. | 0 | 1 |
| **Behavior (total score = 3)** | | |
| Give nutrition counseling or education for hospitalized COVID-19 patients. | 1 | 0 |
| Monitor weight change of hospitalized COVID-19 patients. | 1 | 0 |
| Monitor dietary intake of hospitalized COVID-19 patients. | 1 | 0 |

**Supplementary Table S2**. Adjusted multivariate regression coefficient (β) and 95% Confidence Intervals (95% CI) for length of stay and mortality of hospitalized COVID-19 patients in Indonesia.

The length of stay and mortality of hospitalized COVID-19 patients were defined as the time from the date of first COVID-19 case admitted to the hospital till the end of survey at the end of January 2021. The average length of stay of COVID-19 patients (mild, moderate, severe and critical illness) was obtained from each hospital. The COVID-19 mortality, as total number of death or as case fatality ratio (the number of deaths divided by the number of confirmed case) in each hospital, was derived from the Ministry of Health, Indonesia from National Disaster Management Agency website (https://covid19.bnpb.go.id/).

| Variables | Length of Stay[a,*] | | | | | | | | Mortality[b,*] | p-value |
|---|---|---|---|---|---|---|---|---|---|---|
| | **Mild Symptoms** | **p-value** | **Moderate Symptoms** | **p-value** | **Severe Symptoms** | **p-value** | **Critical illness** | **p-value** | | |
| **Total adherence score** | -0.09 (-0.19, 0.01) | 0.087 | -0.09 (-0.21, 0.04) | 0.180 | -0.15 (-0.33, 0.04) | 0.117 | -0.13 (-0.43, 0.17) | 0.380 | -0.78 (-6.64, 5.09) | 0.791 |
| **Knowledge (total score)** | -0.09 (-0.30, 0.09) | 0.275 | 0.18 (-0.42, 0.06) | 0.130 | -0.29 (-0.64, 0.07) | 0.112 | -0.26 (-0.64, 0.11) | 0.169 | -3.93 (-15.16, 7.30) | 0.487 |
| Guideline awareness | **-0.20 (-0.41, -0.01)** | **0.047** | **-0.32 (-0.56, -0.07)** | **0.012** | **-0.46 (-0.82, -0.09)** | **0.016** | **-0.52 (-0.90, -0.14)** | **0.009** | -2.79 (-9.21, 14.80) | 0.643 |
| Guideline Familiarity | 0.18 (-0.26, 0.61) | 0.425 | 0.28 (-0.27, 0.83) | 0.310 | -0.19 (-0.99, 0.63) | 0.651 | -0.38 (-1.24, 0.48) | 0.383 | **-40.95 (-63.95, -17.95)** | **0.001** |
| **Attitude (total score)** | -0.17 (0.45, 0.11) | 0.230 | -0.24 (-0.58, 0.11) | 0.178 | -0.36 (-0.87, 0.15) | 0.161 | -0.44 (-0.98, 0.99) | 0.107 | -0.58 (-21.94, 10.32) | 0.474 |
| Self-efficacy or confidence | 0.02 (-0.30, 0.35) | 0.884 | -0.08 (-0.48, 0.33) | 0.702 | -0.45 (-1.03, 0.15) | 0.136 | -0.24 (-0.87, 0.40) | 0.462 | 0.92 (-17.84, 19.68) | 0.922 |
| Motivation | -0.27 (-0.70, 0.16) | 0.210 | -0.31 (-0.85, 0.23) | 0.254 | 0.03 (-0.78, 0.84) | 0.935 | -0.042 (-0.90, 0.82) | 0.922 | -11.16 (-36.24, 13.92) | 0.377 |
| **Environmental factor (total score)** | -0.19 (-0.45, 0.07) | 0.151 | 0.02 (-0.31, 0.35) | 0.917 | -0.09 (-0.58, 0.40) | 0.723 | 0.44 (-0.47, 0.56) | 0.865 | 3.60 (-11.63, 18.83) | 0.638 |
| **Nutrition care behavior (total score)** | -0.08 (-0.39, 0.23) | 0.617 | -0.15 (-0.54, 0.24) | 0.439 | -0.15 (-0.73, 0.43) | 0.608 | -0.51 (-1.93, 0.91) | 0.456 | 4.92 (-13.07, 22.92) | 0.586 |

Total adherence score (maximum 28 score) was defined as knowledge (12 questions), attitudes (6 questions), environmental factors (7 questions), and behaviors (3 questions)

*Results were adjusted for age, type of hospital, confirmed cases of covid-19 in each of hospital.

[a] Length of stay was defined from average of length of stay in each hospital

[b] mortality (case fatality) was defined as the number of deaths.
